# Glucose-sensing microRNA-21 disrupts ROS homeostasis and impairs antioxidant responses in cellular glucose variability

**DOI:** 10.1186/s12933-018-0748-2

**Published:** 2018-07-23

**Authors:** Lucia La Sala, Simona Mrakic-Sposta, Stefano Micheloni, Francesco Prattichizzo, Antonio Ceriello

**Affiliations:** 1 Department of Cardiovascular and Dysmetabolic Diseases, IRCCS MultiMedica, Via Fantoli 16/15, 20138 Milan, Italy; 2 Institute of Molecular Bioimaging and Physiology, National Research Council, Segrate, Italy; 3 Institut d’Investigacions Biomèdiques August Pi i Sunyer (IDIBAPS) and Centro de Investigación Biomédica en Red de Diabetes y Enfermedades Metabólicas Asociadas (CIBERDEM), Barcelona, Spain

**Keywords:** miR-21, ROS homeostasis, KRIT1, SOD2, Superoxide anion, Mitochondrial dysfunction, Glucose variability, Oscillating glucose

## Abstract

**Background:**

Antioxidant enzymes play a fundamental role in counteracting oxidative stress induced by high glucose. Although mitochondrial superoxide dismutase (SOD2) is the principal defence against the toxicity of superoxide anions, the mechanism of its inactivation in diabetic subjects is still poorly understood. Recently, microRNA-21 has been associated with diabetes, although its function remains unclear. We sought to explore the mechanism underlying defective SOD2 antioxidant response in HUVECs during exposures to constant high glucose and oscillating glucose (as glucose variability model, GV) and the role of miR-21 in increasing the susceptibility to oxidative stress by disrupting reactive oxygen species (ROS) homeostasis.

**Methods:**

HUVECs exposed for 1 week to constant high glucose and GV were subjected to quantitative electron paramagnetic resonance for ROS measurements. Superoxide anions, SOD2 protein levels and mitochondrial membrane potential (ΔΨm) were also evaluated. Endogenous miR-21 and its putative ROS-homeostatic target genes (KRIT1, FoxO1, NFE2L2 and SOD2) were tested using mimic-miR-21 and quantified by qPCR. Luciferase assays were performed to test miR-21/3′-UTR-SOD2 binding.

**Results:**

We observed upregulation of microRNA-21, overproduction of superoxide anions and total ROS generation, depolarisation of the mitochondrial membrane potential (ΔΨm) and defective SOD2 antioxidant response in HUVECs subjected to constant high glucose and GV exposures. We also found that exogenous mimic-microRNA-21 targeted putative microRNA-21 ROS-homeostatic target genes, e.g., KRIT1, NRF2 and SOD2, which were significantly downregulated. All these effects were reverted by a microRNA-21 inhibitor, which improved SOD2 and KRIT1 expression, reduced the levels of ROS production and ameliorated ΔΨm.

**Conclusions:**

Our data demonstrate the association of microRNA-21 with oscillating and high glucose and early mitochondrial dysfunction. We found that microRNA-21 may promote the suppression of homeostatic signalling that normally limits ROS damage. These data provide novel clues about the inhibition of microRNA-21 as a new therapeutic approach to protect against cellular oxidative injury in glucose variability and diabetes.

**Electronic supplementary material:**

The online version of this article (10.1186/s12933-018-0748-2) contains supplementary material, which is available to authorized users.

## Introduction

Type 2 diabetes (T2D) is a complex metabolic disease associated with insulin resistance, obesity and the development of cardiovascular disorders (micro- and macrovascular), whose central factors are involved in the occurrence of high and prolonged cellular oxidative stress (Ox-S) [1]. The fluctuation of blood glucose as assessed by glycaemic variability (GV), a phenomenon that occurs daily in diabetic subjects, can induce functional and structural alterations in the endothelium [2, 3]. Since GV is considered a strong predictor of the elevated risk of developing vascular complications [4, 5], even in non-diabetic subjects [6], it has attracted a great deal attention in clinical practice. Thus, development of a cellular model to better understand the molecular mechanisms of GV induction and how it is regulated represents a challenge. Recently, in our previous in vitro studies, we showed that GV may have more deleterious effects on endothelial cells than high glucose and that it may activate several metabolic pathways directly responsible for cellular damage [7, 8]. The mechanisms responsible for endothelial damage are closely related to hyperglycaemia-induced mitochondrial reactive oxygen species (ROS) overproduction, resulting in defective ROS homeostasis and inactivation of antioxidant responses, which are responsible for controlling the rate of radicals produced under stress conditions. ROS are a natural by-product of electron transport chain activity arising from the reduction of molecular oxygen or oxidation of water to yield products such as superoxide anion (O_2_·−), hydrogen peroxide (H_2_O_2_), and hydroxyl radical (OH·); their progressive accumulation from endogenous metabolic processes or impaired removal can disrupt the redox balance, leading to DNA damage [9].

The protective mechanisms induced by hyperglycaemic damage involve antioxidant enzymes that balance ROS production. Recently, we observed impaired expression of manganese-superoxide dismutase 2 (SOD2) in HUVECs exposed to a GV model [8], suggesting the damaging effects of hyperglycaemia on the antioxidant defence system. In addition, animal models have shown that the lack of SOD2 expression was associated with dilated ventricular cardiomyopathies and increasing susceptibility to Ox-S [10]. Moreover, human polymorphisms of SOD2 have been associated with cardiomyopathy and diabetic microvascular complications [11–13]. Although SOD2 is one of the numerous defence enzymes counteracting oxidant injury, particularly mitochondrial damage [14], the molecular and cellular events mediating the impaired redox state, and thus the SOD2-antioxidant response, remain elusive.

Epigenetics has emerged as new regulatory mechanism to control gene expression. MicroRNAs (miRs), as major contributors to epigenetic gene silencing, are endogenous ~ 23 nt long RNAs repressing the expression of complementary messenger RNAs by pairing to the untranslated region (3′-UTR) [15]. miRs are largely found in tissues, cells and biological fluids, suggesting they have a potential role as a key diagnostic tool. Importantly, miRs have key roles in metabolism during development and disease [16]. Recently, human studies have implicated miR-21 in proliferative diabetic retinopathy and cardiovascular disease [17, 18], but it is still unclear whether and how miR-21 is linked to hyperglycaemia.

In this study, we aimed to demonstrate that GV and constant high glucose induce the downregulation of a ROS homeostasis regulator, Krev/Rap1 interaction trapped-1 (KRIT1), and the defective SOD2 antioxidant response via miR-21 activation.

## Materials and methods

### Cells and cell culture

Primary pooled human umbilical vein endothelial cells (HUVECs) and growth factors were purchased from Lonza (CC-2519, Lonza Bioresearch LBS, Basel, Switzerland). Cells were cultured in endothelial basal medium (EBM-2), supplemented with low foetal bovine serum (2%), hydrocortisone (1 µg/mL), basal fibroblastic growth factor (bFGF, 5 ng/mL), epidermal growth factor (rhEGF, 5 ng/mL), heparin (0.75 units/mL) and gentamicin/amphotericin (GA-1000, 0.1%) in a humidified incubator with 5% carbon dioxide added.

#### Experimental design of glucose variability

First, 2 × 10^5^ primary HUVECs/well at p4 were exposed to three different glucose concentrations for 1 week: (1) normal glucose (NG, 5 mmol/L), (2) oscillating glucose (OG, 5/25 mmol/L), and (3) high glucose (HG, 25 mmol/L) [19]. Oscillating glucose conditions (GV experiments) were obtained by changing the glucose concentration (from 5 to 25 mmol/L) every day for 7 days [19]. In addition, cells were maintained for 2 days more to perform transfections.

### Cellular induction of oxidative stress

Oxidative stress generation was induced in 24 h-cultured HUVECs exposed to normal glucose (5 mmol/l) and hydrogen peroxide (H_2_O_2_, 500 μM for 6 h). As an antioxidant agent, alpha-lipoic acid (aLA) purchased from Sigma-Aldrich (St. Louis, MO, USA) was used, and the drug was administered each day, as described in modified protocols [7].

### RNA extraction and real-time PCR analysis

Total RNA was extracted from HUVECs using an RNA purification kit (NorgenBiotek, Thorold, ON, Canada) following the manufacturer’s instructions. First, 1–2 micrograms of total RNA was reverse transcribed using SuperScript VILO transcriptase (Thermo Fisher, Monza, IT). Real-time q-PCR was performed using a QuantStudio 6 flex (Applied Biosystems, Foster City, CA, USA) detection system with SybrGreen reagent (TaKaRa Bio Company, Clontech, Mountain View, CA, USA). Specific primers are listed in Table 1.

**Table 1:** Primer sequences for qRT-PCR analysis

Gene	Sequence
KRIT1	*f*-ACTGTTACTCAAGCCACCACA
	*r*-ATGCTAGGGCCCAAAAGTAAT
FOXO1	*f*-TGCATTTCGCTACCCGAGTT
	*r*-GTGGCTGACAAGACTTAACTCAA
NFE2L2	*f*-AGGTTGCCCACATTCCCAAA
	*r*-AGTGACTGAAACGTAGCCGAA
SOD2	*f*-GGCCTACGTGAACAACCTGA
	*r*-CAGGACGTTATCTTGCTGGG
SOD1	*f*-GCTGGTTTGCGTCGTAGTCT
	*r*-CCACACCTTCACTGGTCCAT
CAT	*f*-CTTCGACCCAAGCAACATGC
	*r*-TAATTGGGTCCCAGGCGATG
GPx-1	*f*-CCCAGTCGGTGTATGCCTTC
	*r*-AGCATGAAGTTGGGCTCGAA
ACTIN-b	*f*-CAGCCATGTACGTTGCTATCCAGG
	*r*-AGGTCCAGACGCAGGATGGCATG

All *q*-*PCR* results were normalized to actin (ACTIN-b) for gene expression, and RNU6 (assay ID 001973, Applied Biosystems, Life Technologies, Grand Island, NY, USA) was used as an endogenous miRNA control. Data were obtained as Ct values, and the 2^−ΔCt^ method was used in the analysis.

### Determination of ROS by EPR

Reactive oxygen species generation was detected in HUVEC culture media under glucose exposure by electron paramagnetic resonance (EPR) spectroscopy (EPR spectrometer, Bruker, Karlsruhe, Germany), operating at the common X-Band microwave frequency (9.8 GHz). Extracellular fluids were incubated with 1 mM CMH (1-hydroxy-3-methoxycarbonyl-2,2,5,5-tetramethylpyrroline) probe prepared in buffer [Krebs-Hepes buffer (KHB) containing 25 μM deferoxamine methane-sulfonate salt (DF) chelating agent and 5 μM sodium diethyldiothio-carbamate trihydrate (DETC) at pH 7.4]. Spectra were recorded and analysed by standard software (Win EPR 2.11, Bruker). Data were normalized to the number of viable cells.

### Mitochondrial membrane potential (ΔΨm)

JC-1 (5,5′,6,6′-tetrachloro-1,1′,3,3′-tetra-ethylbenzimidazol-carbocyanine iodide), a lipophilic cationic dye that can selectively enter into mitochondria, was purchased from Cayman Chemicals (Ann Arbor, MI, USA) and assessed by fluorescence microscopy (Axio Vision, Zeiss, München, Germany) as described in the manufacturer’s instructions.

### Analysis of mitochondrial superoxide production in HUVECs

#### MitoSOX Red-mitochondrial superoxide production

Mitochondrial superoxide production was measured as the change in fluorescence intensity of MitoSOX Red (λex/λem = 488/580) (Thermo Fisher, Monza, IT) in response to oscillating glucose (OG), to high glucose (HG) and to anti-miR-21 (50 nmol/L) using fluorescence microscopy (Zeiss). Briefly, to avoid the intrinsic cytotoxicity of the dye, the cells were incubated at 37 °C for 10 min with 2 μmol/L MitoSOX Red and diluted in HBSS with Ca^2+^/Mg^2+^. Then, cells were washed with PBS supplemented with 2% FBS and fixed in 2% paraformaldehyde (PFA) for 10 min [20]. Nuclei were stained with DAPI (blue). The change in superoxide production was quantified as the difference in MitoSOX Red fluorescence intensity among controls and treated samples.

#### FACS analysis of mitochondrial superoxide production

FACS analysis was performed using 2 μmol/L MitoSOX Red at 37 °C for 10 min. Briefly, the cells were collected by trypsinisation and washed with PBS supplemented with 2% FBS, fixed in 2% paraformaldehyde (PFA) for 10 min and re-suspended in 500 μL of PBS with 2% FBS. Using FACS, MitoSOX Red was excited at 488, and fluorescence emission at 580 was measured. Superoxide was detected with a FACSCanto cytometer (BD Biosciences) on the FL1 channel and analysed with Flowlogic software (Milteny Biotec).

### miRNA functional studies

#### Endogenous expression and gain- and loss-of function of miR-21

miR-21 expression was examined with the TaqMan MicroRNA Assay Kit (Applied Biosystems, Life Technologies, Grand Island, NY, USA). MultiScribe Reverse Transcriptase was used for RT-PCR, and TaqMan primers for hsa-miR-21 (assay ID 000397) were used to monitor endogenous miRNA expression. RNU6 (assay ID 001973) was used as an endogenous miRNA control (all purchased from Applied Biosystems). Anti-miR™ miRNA-21 inhibitor (MH10206), an antisense miR-21, scrambled Anti-miR™ miRNA inhibitor negative control (AM17010), mirVana-mimic miR-21 and its negative control were purchased from Ambion (Foster City, CA, USA). Transfections of miRNA inhibitors were performed at least three times in triplicate using INTERFERin^®^ transfection reagent according to the manufacturer’s protocol (POLYPLUS-transfection, NY, USA).

#### HUVEC co-transfection for functional assay

Primary HUVECs at 5 × 10^4^ cells in passage 4 (p4) were transiently co-transfected with a 3′-UTR-SOD2 expression vector with a firefly luciferase reporter (Origene, MD, USA) and miRNA-21 mimic sequence. We used the 3′-UTR-SOD2 expression vector whose cloning region contained the entire 762 bp SOD2 3′-UTR variant 1 (NM_000636), in line with bioinformatics prediction analysis, to identify the miR-21 targets. JetPRIME co-transfection reagent was used for the experiments following the manufacturer’s instructions (POLYPLUS). Briefly, controls cells were transfected with 500 ng of empty vector (pMIR) alone and with pMIR with 50 nmol/L of miRNA-21 mimic sequence. The thymidine kinase promoter-Renilla luciferase reporter plasmid (pRL-TK) (10 ng) was used as an internal control for transfection efficiency. Cells were processed for lysis and collected 48 h after co-transfection. Renilla and luciferase activity of total cell lysates was measured using a luciferase reporter assay kit (Dual Glo™ Luciferase Reporter System, Promega, USA) with a GloMax luminometer (Promega). Luciferase values were normalized by calculating RLU/μg protein.

The luciferase assay for KRIT1 was omitted, as it was previously performed by others [21]. Moreover, luciferase assays for FOXO1 and NFE2L2 were omitted, as we had found normal mRNA levels for FOXO1 and NFE2L2 in HUVECs transfected with anti-miR-21 (data not shown). mRNA expression of NFE2L2 was unchanged in OG and HG conditions. The level of the miR-21 inhibition was drastically reduced in the presence of the anti-miR-21, as determined by q-PCR (Fig. 6).

### SDS-PAGE

For western blot analysis, HUVECs were lysed in RIPA buffer (Sigma-Aldrich) with 10% protease and 1% phosphatase inhibitors (Sigma-Aldrich). Protein content was determined using the Bradford assay (Sigma-Aldrich), and 50 µg of lysates was separated by electrophoresis using 4–12% PAGE gels (Lonza) and transferred onto a PVDF membrane (Perkin Elmer, Waltham, MA, USA). After membranes were blocked with 5% non-fat dried milk or 5% bovine serum albumin, they were incubated with primary antibodies overnight at 4 °C: phospho-NRF2-Ser40 (1:1000) and NRF2 (1:1000) (Abcam, ab76026 and ab137550), phospho-ERK1/2-Thr202/Tyr204 (1:1000) and ERK1/2 (1:1000) (9101 and 9102, Cell Signaling), and SOD2 (1:500; sc-133134, Santa Cruz Biotechnology, CA, USA). Blots were revealed by LI-COR ECL Reagent and by a C-DiGit Blot scanner (LI-COR Biosciences) after incubation with appropriate secondary horseradish peroxidase-conjugated IgG antibodies (GE Healthcare Europe GmbH, Milan, Italy) at a 1:3000 dilution for 1 h at room temperature. Proteins were quantified using Image Studio software (http://www.licor.com). Human ACTIN-b (1:1000) (Sigma-Aldrich) was used as a loading control.

### KRIT1 fluorescence microscopy

Cells were fixed in 4% paraformaldehyde for 10 min and permeabilised with 0.1% Triton X-100 for 5 min. After blocking with 5% donkey serum/PBS, the cells were incubated with anti-human KRIT1 (Abcam, ab196025) antibody at 4 °C overnight. Then, cells were incubated with Alexa Fluor 488 anti-rabbit IgG (Invitrogen, Carlsbad, CA, USA) as secondary antibodies at room temperature for 60 min. DAPI (Invitrogen) was used for nuclei counterstaining.

### Statistical analysis

Results are expressed as mean ± SEM. Differences between groups were evaluated using one-way ANOVA, followed by the Tukey’s post hoc test. All reported p-values were two-sides. A p-value of < 0.05 was considered statistically significant. Statistical analyses were performed with GraphPrism6.0 (http://www.graphpad.com).

## Results

### Intracellular miR-21 is induced by oscillating (OG) and high glucose (HG), affecting ROS generation

**Fig. 1:**
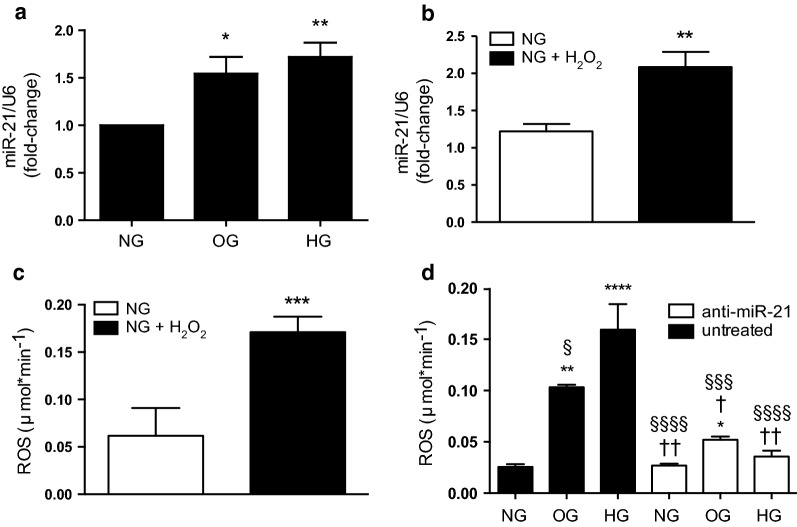
miR-21 expression in HUVECs is increased in oscillating (OG) and high stable glucose (HG) conditions. **a** miR-21 expression was normalised to RNU6 expression using the comparative Ct method. Experiments were performed in duplicate and repeated 6 times. **b** To assess the relationship between miR-21 and oxidative stress generation, we demonstrated that in 24 h-cultured HUVECs exposed to normal glucose (5 mmol/L) and hydrogen peroxide (H_2_O_2_, 500 μM for 6 h), intracellular miR-21 and **c** extracellular ROS release (measured by EPR instruments); **d** ROS release in HUVECs, with or without an anti-miR-21 inhibitor, were significantly increased in OG and HG conditions (p < 0.01 and p < 0.001, respectively), demonstrating a potential role for miR-21 in oxidative stress. miR-21 relative expression was normalized to U6 levels. One-way ANOVA followed by Tukey’s post hoc test. *p < 0.05 vs NG, **p < 0.01 vs NG, ****p < 0.0001 vs NG; ^†^p < 0.05 vs OG, ^††^p < 0.01 vs OG; ^§§§^p < 0.001 and ^§§§§^p < 0.0001 vs HG

In primary HUVECs, in both OG and HG conditions, miR-21 expression levels were increased with respect to controls (p < 0.05 OG vs NG, p < 0.01 HG vs NG) (Fig. 1a). Recently, miRNAs have been shown to be modulators of cellular stress. To dissect the relative contribution of miR-21 towards ROS production, we performed oxidative stress induction experiments in 24 h-cultured HUVECs (Fig. 1b, c); the results showed increased miR-21 levels during exposure to a prooxidant agent, such as hydrogen peroxide (t-test, p < 0.01, Fig. 1b). We measured ROS absolute concentrations in the culture media of HUVECs by electron paramagnetic resonance (EPR) to demonstrate their “instantaneous” presence, and their ROS levels were also found to be increased during hydrogen peroxide treatment (t-test, p < 0.001, Fig. 1c).

To define the effects of miR-21 on endothelial ROS inductions, we silenced miR-21 expression using the Anti-miR™ miRNA miR-21 inhibitor. We transfected 50 nmol/L of anti-miR-21 into HUVECs cultured for 1 week in NG, OG and HG. Quantitative analysis of extracellular ROS showed significant overexpression in both OG and HG (p < 0.01 and p < 0.001 vs NG, respectively) (Fig. 1d). When miR-21 was silenced, the overproduction of ROS was significantly reduced in both OG and HG (p < 0.05 vs OG and p < 0.01 vs HG, respectively), and the phenotype appeared to revert. Furthermore, under fluorescence microscopy, no morphological differences were observed between cells with miR-21 silencing, nor were there any significant changes in cellular viability (Additional file 1: Figure S1a). The transfection efficiencies of miR-21 were very high, as shown by q-PCR, and the intracellular levels of miR-21 were significantly reduced (**p < 0.01, NG + anti-miR-21 vs NG; ^†††^p < 0.001, OG + anti-miR-21 vs OG; ^§§§^p < 0.001 HG + anti-miR-21 vs HG; Additional file 1: Figure S1). No differences in NG compared with scrambled miRNA transfection were found (data not shown).

### Oscillating and high glucose conditions induce accumulation of superoxide and disrupt the mitochondrial membrane potential

**Fig. 2:**
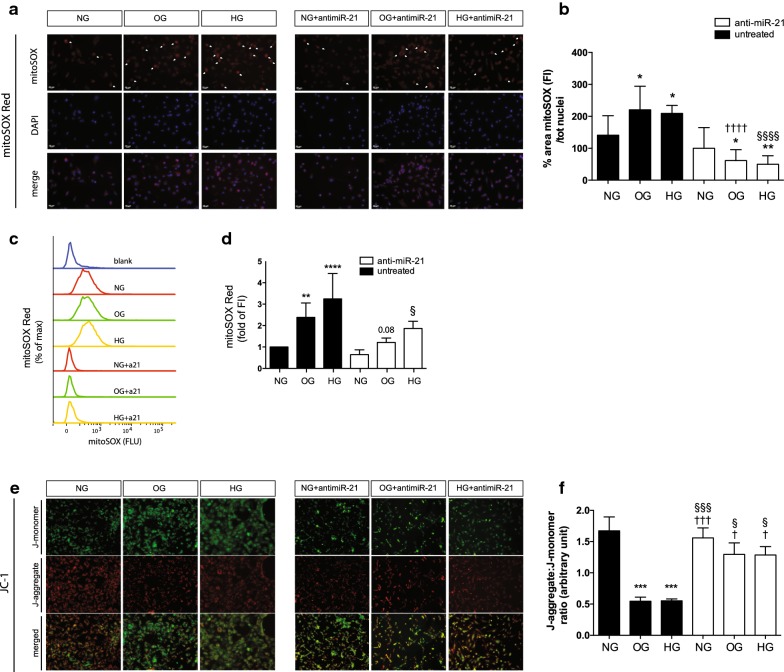
Silencing miR-21 reduces ROS and ameliorates mitochondrial membrane potential (ΔΨm) in oscillating (OG) and high stable glucose (HG) conditions. **a** Representative images of oxidized MitoSOX Red staining using fluorescence microscopy. Cells imaged by fluorescence microscope using a rhodamine filter (peak excitation 547 nm, emission filter 580 nm long pass). Arrows show the major fluorescence intensity peak for each image. **b** Quantification of MitoSOX images measured the % of MitoSOX fluorescence intensity area/count of nuclei (DAPI) *per* field (at least n = 6 *per* condition). The red intensity (arbitrary fluorescence units equivalent to mitochondrial superoxide production) was quantified, integrated, and normalised to integrated DAPI intensity (equivalent to cell number) using ImageJ. Scale bar, 20 μm. *p < 0.05 and **p < 0.01 versus NG, ^††††^p < 0.0001 vs OG and ^§§§§^p < 0.0001 vs HG. **c** Selective detection of mitochondrial superoxide anion using MitoSOX Red staining by FACS analysis (n = 3 set experiments) expressed as a % of the max fluorescence intensity. **d** Densitometry of FACS as a fold change of the fluorescence intensity. **p < 0.01 and ****p < 0.0001 vs NG, ^§^p < 0.05 vs HG. **e** Representative images of fluorescence microscopy show mitochondrial membrane potential (ΔΨm) changes. Red staining indicates JC-1 aggregates in control cells (NG), revealing intact mitochondria, and the formation of monomers (green staining) shows dissipation of ΔΨm. A decrease in monomers and increase in aggregates (red staining) indicate the ability of anti-miR-21 to protect the mitochondria. **f** The graph under the images indicates the ratio of JC-1 aggregates to JC-1 monomers calculated by ImageJ software (http://www.imagej.nih.gov, ImageJ, NIH, Bethesda, MD, USA). The results are expressed as mean (± SEM) of the ratio of arbitrary unit intensity, n = 6 and ***p < 0.001 vs NG; ^†^p < 0.05 vs OG, ^†††^p < 0.001 vs OG; ^§§§^p < 0.001 vs HG. Symbols over the bars refer to differences between the conditions shown under the bars

Superoxide anion is a primary ROS and a precursor of many secondary ROS, e.g., hydrogen peroxide, hydroxyl radical, hypochlorous acid, and hydroperoxyl radical. Although the major site of superoxide production within the mitochondria is still controversial, most evidence indicates that superoxide is derived from respiratory chain complex I and complex III. High levels of ROS may also be derived from cellular organelles (outside mitochondria), including oxygen radicals from the peroxisomal β-oxidation of fatty acids [22], NAD(P)H oxidase [23], xanthine oxidase, arachidonic acid metabolism, microsomal P-450 enzymes [24] and the pro-oxidant haeme molecule [25], adding a further layer of complexity and regulation. The release of mitochondrial-specific superoxide anions under OG and HG conditions was analysed by immunofluorescence (Fig. 2a, b) and by FACS analysis (Fig. 2c, d) with MitoSOX Red staining reagent. The production of superoxide anion increased in both conditions. miR-21 knockdown inhibited MitoSOX Red intensity compared with that of the static condition (Fig. 2a, b). In addition, to explore the close link between mitochondrial function and high glucose, we evaluated the mitochondrial membrane potential (ΔΨm or MMP) in miR-21-expressing cells and compared it with that in miR-21-deficient effect (or anti–miR-21 treatment) (Fig. 2e, f). The selective mitochondrial probe JC-1 was used; it accumulates in the mitochondrial membrane and emits a red fluorescent signal when ΔΨm is high and a green fluorescent signal when ΔΨm is low [26, 27]. OG and HG induced severe changes in ΔΨm, which collapsed in both OG and HG (p < 0.001; Fig. 2f), while JC-1 aggregates remained in the cytoplasm in a green monomeric form (Fig. 2e). In silenced miR-21 cells, the ratio between aggregated and monomeric forms indicated the enhanced ΔΨm in OG (p < 0.05 vs OG with no anti-miR-21) and HG (p < 0.05 vs HG with no anti-miR-21), corroborating the anti-miR-21 therapeutic actions on ROS inhibition and the restoration of mitochondrial function. Collectively, these data highlighted a critical role of miR-21 in the disruption of mitochondrial homeostasis.

### KRIT1, FoxO1, NFE2L2 and SOD2 are putative targets of miR-21

Because a microRNA can affect a complete network of genes either directly or indirectly, we analysed some ROS-homeostatic target genes, including KRIT1 and FoxO1, to explore the miR-21 function and the antioxidant response it may trigger by analysing the NFE2L2 and SOD2 genes. For this purpose, we performed a computational analysis using three in silico prediction tools available online (DIANA-microT-CDS, http://diana.imis.athena-innovation.gr [28]; MiRTarBase, http://mirtarbase.mbc.nctu.ed-u.tw [29]; and TargetScan 7.1, http://www.targetscan.org [30]) for KRIT1, FoxO1, NFE2L2 and SOD2. These tools identified three binding sites of human miR-21 on the 3′-UTR of the KRIT1 gene, predictions that have been confirmed in BC1 cell lines using high-throughput sequencing and crosslinking-immunoprecipitation (HITS-CLIP) data sets [31].

**Fig. 3:**
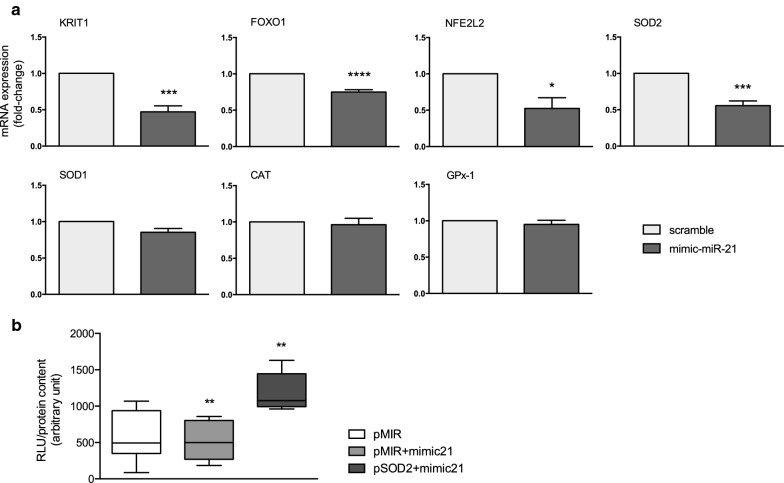
Functional assay. **a** Gain-of-function assay using an exogenous mimic-miR-21 was used to perform an initial screen of miR-21 target genes. q-PCR analyses of KRIT1, FOXO1, NEFL2L, SOD2, SOD1, CAT and GPx-1 mRNA relative expression levels were performed in HUVECs cultured in normal glucose (5 mmol/L) for 24 h and compared to scrambled control. A two-tailed t-test was applied for statistical analysis. *p < 0.05, ***p < 0.001, both vs scrambled control. **b** Luciferase assays tested the binding between 3′-UTR SOD2 and miR-21. The luciferase data are expressed as a ratio between firefly and renilla luciferase to normalize for the transfection variability between each sample and subsequently normalized against lysate protein levels to account for differences in the total protein concentration. Measurements were carried out in quadruplicate for three independent experiments. **p < 0.01, ANOVA 1-way, followed by Tukey’s multiple comparison test

FoxO1, which controls a variety of target genes, including antioxidant genes, was predicted only using the DIANA-microT-CDS tool, whereas NFE2L2 and SOD2 showed no binding sites with any of the tools used, suggesting an ancillary effect. Next, to validate the predictions and evaluate the putative miR-21 target genes, we transfected HUVECs previously exposed for 24 h to normal glucose to the exogenous mimic-miR21 sequence (50 nmol/L). Among the genes tested during the upregulation of miR-21 activity, the mRNA levels of KRIT1, FoxO1, NFE2L2 and SOD2 were significantly downregulated (p < 0.001, p < 0.0001, p < 0.05 and p < 0.001, respectively) (Fig. 3a). In addition, we found unchanged levels for other antioxidant defence genes, including SOD1, CAT and GPx-1 (Fig. 3a).

### SOD2 is not a direct target of miR-21 in HUVECs

The miRNA–mRNA base pairing typically occurs at the ‘seed’ (2–7 nucleotides) region of the miRNA [32]. Often the interactions follow different rules for target recognition that are not foreseen at all by current prediction tools, which are based on canonical perfect base pairing to the seed sequence. Thus, we used RNAhybrid [33], which is able to show the free energy release by binding and thus improves the confidence of predictions. Certainly, the ability of miRs to repress mRNA targets is dependent on free energy of binding of the first eight nucleotides to the 5′ region of miRNAs [34]. We found a binding site for miR-21 to the 3′-UTR of SOD2 in position 193. Although predictions showed a highly favourable minimum free energy score of ΔG (Gibbs free energy) = − 18 kcal/mol, the luciferase assay did not show any binding (Fig. 3b).

### Knockdown of miR-21 enhances KRIT1 and SOD2 expression levels during oscillating and high glucose exposures

Given the importance of KRIT1 in the homeostasis of ROS, we hypothesized that miR-21 might influence the cellular susceptibility to Ox-S by modulating KRIT1 and affecting SOD2 expression. The potential roles of miR-21 were determined by silencing using the miR-21 Anti-miR™ miRNA Inhibitor. Interestingly, following 1 week of glucose exposure, the mRNA levels of KRIT1 were reduced (p < 0.05 for both OG and HG), while SOD2 exhibited a trend of reduced levels in both OG and HG, suggesting a defective antioxidant response.

**Fig. 4:**
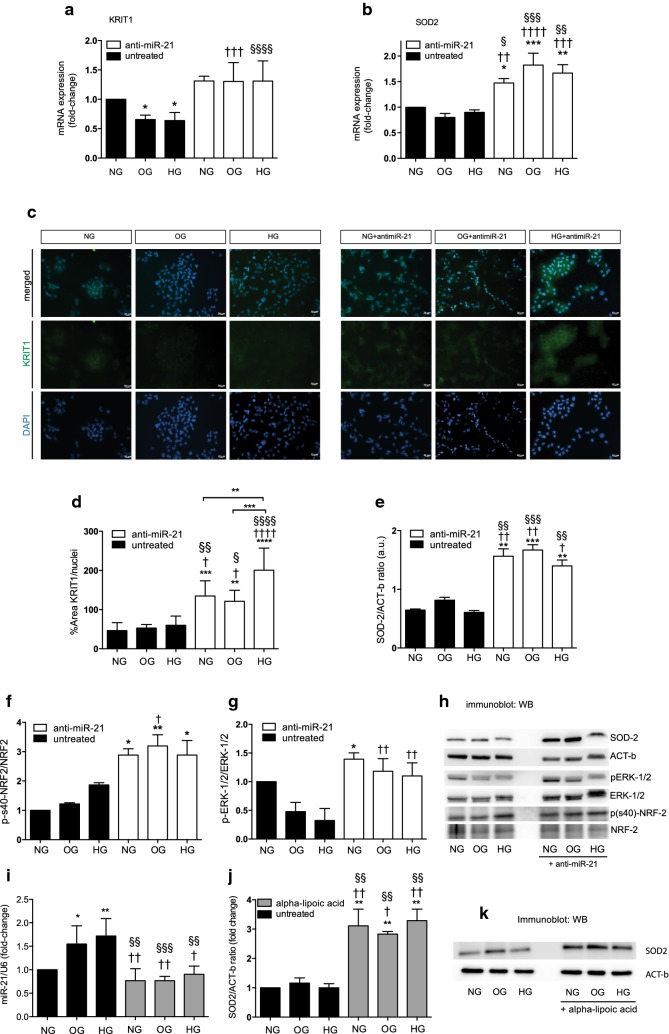
Effects of intracellular miR-21 knockdown in ameliorating ROS homeostasis in increasing the antioxidant response in HUVECs. **a** KRIT1 mRNA expression levels were reduced in OG and HG, whereas anti-miR-21 reversed this effect. **b** SOD2 mRNA was unchanged during glucose concentration changes, but the level was increased during miR-21 silencing. **c** Endogenous KRIT1 staining was reduced in OG and HG. Silencing miR-21 increased KRIT1 fluorescence intensity. Fluorescence images are representative of n = 6 *per* experiments. Original magnification ×20; scale bar, 20 μm. **d** Densitometry of KRIT1 images measured by the ratio of the % area of positive staining and nuclei count (DAPI) *per* field (at least n = 6 *per* condition). **e**–**h** Whole cell lysates densitometry of (E) SOD2 protein expression levels, phosphorylation of **f** serine-40 on NRF2 and **g** ERK1/2. Normalisation was to actin-beta (ACT-b) and to total amounts of ERK1/2 and NRF2, respectively for phosphorylated status. **h** Representative immunoblots of HUVECs under NG, OG and HG, before and after transfection with an anti-miR-21 inhibitor. **i**–**k** miR-21 expression levels during administration of ROS scavenger, alpha lipoic acid (aLA), that reduced miR-21 expression and **j** conversely increased SOD2 protein levels; **k** representative immunoblots of SOD2 normalised to ACT-b levels. Data are mean (± SEM), *p < 0.05, **p < 0.01, and ***p < 0.001 vs control; ^†^p < 0.05, ^††^p < 0.01, ^†††^p < 0.001 and ^††††^p < 0.0001 vs OG, ^§^p < 0.05, ^§§^p < 0.01, ^§§§^p < 0.001 and ^§§§§^p < 0.0001 vs HG

However, the knockdown of miR-21 significantly increased these mRNAs containing miR-21 target sequences (Fig. 4a, b). These results were corroborated by assessing the protein levels through immunofluorescence of KRIT1 (Fig. 4c, d) and western blot analysis of SOD2 (Fig. 4e, h); miR-21 silencing restored the protein levels for both. These findings suggest that loss of miR-21 expression surprisingly results in reduced ROS production and in an increased SOD2 antioxidant response. Given that SOD2 is regulated by a plethora of antioxidant gene promoters, including NFEL2L (also known as NRF2), which is downregulated in several diabetes models, we attempted to verify whether NRF2 could modulate SOD2 under miR-21 induction during OG and HG exposures. We demonstrated that the NRF2 form phosphorylated on serine 40 is upregulated in miR-21 knockdown cells (ANOVA, p = 0.0037, Fig. 3f). In addition, aLA administration reduced the miR-21 levels and increased the protein levels of SOD2 (Fig. 4i–k), providing proof of the antioxidant features of the anti-miR-21 inhibitor. Our results show strong evidence of the pleiotropic effects of miR-21 and highlight for the first time its role in the activation of antioxidant response genes in HUVECs.

To test the hypothesis that ancillary mechanisms might be involved in SOD2 transcriptional activation, such as the role of certain kinases that could be post-translationally modulated, we investigated the involvement of ROS-dependent kinases dependent on activation of NRF2. In this context, extracellular signal-regulated kinase (ERK1/2) and its phosphorylated form were evaluated. ERK1/2 was shown to be activated by ROS [35] and required for NRF2 nuclear localization [36]. Phosphorylation was reduced in miR-21-expressing cells, whereas silencing enhanced ERK1/2 activation (Fig. 4g). In addition, to further demonstrate the role of miR-21 in Ox-S, we measured miR-21 and SOD2 levels during treatment with a pro-oxidant agent, hydrogen peroxide, demonstrating the sensitivity of miR-21 expression in an oxidative context, hyperglycaemia-induced.

### Computational analysis of miR-21's putative and validated signalling pathways

**Fig. 5:**
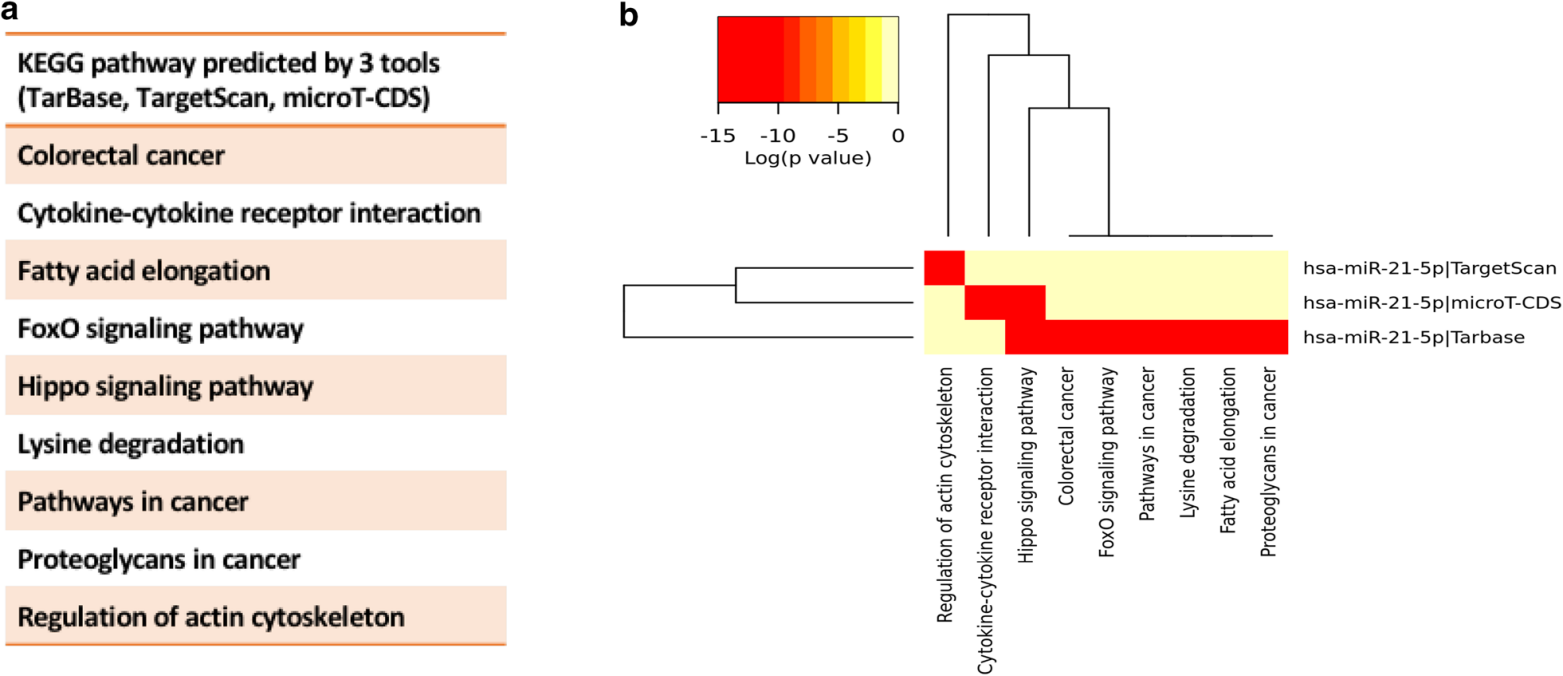
Predicted miR-21 pathways. Targeted pathway clusters/heatmap; p-value threshold 0.05; enrichment analysis: hypergeometric distribution

**Fig. 6:**
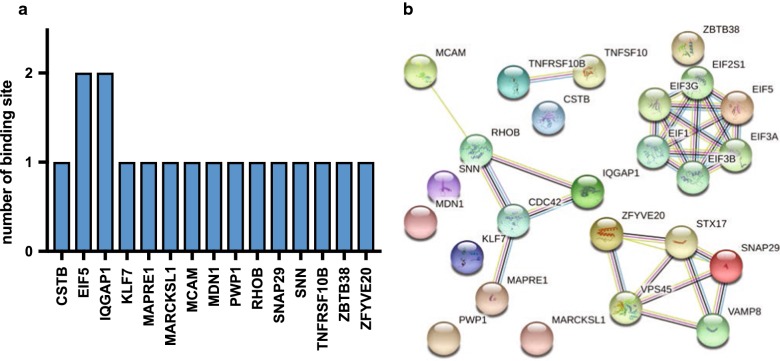
Experimentally validated target genes of miR-21 and related pathways in HUVECs. **a** Number of miR-21 binding sites specifically evaluated in HUVECs by the extrapolation of experimentally supported (direct method: HITS-CLIP) [60] miRNA targets from DIANA-TarBase v8.0. **b** Analysis of target (proteins) network using STRING v10.5 (https://string-db.org): green line—neighbourhood evidence; blue line—co-occurrence evidence; purple line—experimental evidence; light blue line—database evidence; black line—co-expression evidence

Identification of target genes is crucial for characterizing the functions of miRNAs. First, we performed a computational target prediction analysis using the online tool DIANA-miRPath v3.0 (http:/microrna.gr/miRPathv3) with the intersection of 3 tools, TargetScan, microT-CDS and TarBase. The results are shown (Fig. 5) with a heatmap, and the pathways are clustered based on significance levels. Importantly, we extrapolated several experimentally supported miRNA targets, and we integrated the analysis with their signalling pathways, which were specifically evaluated in HUVECs from DIANA-TarBase v8.0 [37] (Table 2; Fig. 6a), with prediction score between 0.794 and 0.451. Functional interactions in biological functions require co-expression of proteins. To elucidate the network of co-expressed targets, we extracted the validated target genes with co-expression coefficients higher than 0.6 from Search Tool for the Retrieval of Interacting Genes (STRING) available online (Fig. 6b).

**Table 2:** Experimentally validated miRNA:mRNA targets in HUVECs

Gene	Binding location	Regulation	Pathways/functions
CSTB	chr21:43774214–43774239	Down	Immune system (reactome)
EIF5	chr14:103342859–103342887; chr14:103336755–103336777	Down	RNA transport (KEGG) Gene expression (reactome)
IQGAP1	chr15:90492649–90492672; chr15:90482260–90482275	Down	Proteoglycans in cancer (KEGG) Adherence junction (KEGG) Cell–cell communication (reactome)
KLF7	chr2:207088518–207088545	Down	Adipogenesis
MAPRE1	chr20:32833787–32833796	Down	Organelle (KEGG)
MARCKSL1	chr1:32334564–32334592	Down	Fc-gamma R-mediated
MCAM	chr11:119310410–119310423	Down	Adhesion
MDN1	chr6:89718909–89718936	Down	Ribosome biogenesis (KEGG)
PWP1	chr12:107688737–107688759	Down	–
RHOB	chr2:20449368–20449389	Down	Axon guidance (KEGG)
SNAP29	chr22:20890084–20890104	Down	Membrane trafficking (reactome)
SNN*	chr16:11678992–11679013	Down	–
TNFRSF10B	chr8:23020409–23020435	Down	p53 signalling pathway (KEGG)
ZBTB38	chr3:141444493–141444514	Down	–
ZFYVE20 (also RBSN)	chr3:15096210–15096236	Down	Endocytosis (KEGG)

For * SNN gene, no data are reported, but SNN interacts with YWHAZ and YWHAB genes of both “Hippo signalling pathway” (not reported in table) and “Cell cycle” of the related predicted KEGG pathways

## Discussion

In the present work, for the first time we examined the complex regulation of the defence system damaged by oscillating and high glucose, as well as the role of miR-21 in the damaging effects of GV and chronic hyperglycaemia. Specifically, we focused on the antioxidant response by SOD2, which is disrupted by miR-21 induction and coupled with the mitochondrial dysfunction. In this context, the mechanism of SOD2 dysregulation is not completely known, but it could be dependent on the defective activation of KRIT1 homeostatic pathways.

There is clear evidence that GV is associated with increased oxidative stress generation accelerating processes that contribute to cellular dysfunction. In vessels, to maintain homeostasis, many cellular and plasmatic defensive mechanisms normally counteract damage in cells and tissues. In the diabetic *milieu*, changes in lipid composition and insulin resistance interfere with these protective mechanisms, promoting tissue injury. However, the reduced capacity to maintain appropriate ROS steady-state levels and enhanced susceptibility to oxidative switch are likely due to concurrent defective ROS scavenging and decreased mitochondrial function. A better understanding of the molecular mechanisms underpinning ROS homeostatic defects will improve current therapeutic options.

Recent evidence has shown that miRNAs have a key role in diabetic disorders, being under the control of various metabolic stimuli, including glucose [38]. Diabetes significantly alters the expression of miRs in circulating endothelial microparticles (MPs), which has implications in vascular health [39] and in prediabetes. In addition, the endothelial miR-126-3p content correlates with markers of endothelial inflammation, such as VCAM-1, plasma antioxidant capacity, and MPs [40].

As reported in our previous work, miRNAs could be important modulators of the antioxidant pathways, since defective antioxidant responses in the GV model seem to be the major cause of cellular damage [8].

miR-21 is encoded by the MIR21 gene, which is located on the plus strand of chromosome 17q23.2 within the coding gene TMEM49 (also called vacuole membrane protein, VMP1) [41]. Despite being located in the 3′-UTR region of a coding gene in the direction of transcription (UCSC genome browser http://genome.ucsc.edu), the MIR21gene has its own promoter regions and forms an ~ 3433-nt long primary transcript of miR-21 (known as pri-miR-21), which is independently transcribed [42]. miR-21 levels are increased selectively in fibroblasts of the failing heart, controlling interstitial fibrosis and cardiac hypertrophy [18], and in atherosclerotic plaques, contributing to the acceleration of VSMC growth [43] and kidney fibrosis [44]. In addition, miR-21 exerts its deleterious actions in myocardial ischaemic-reperfused mice [45], as well as in diabetic nephropathy [38], and in the appearance of insulin resistance (IR) [38]. miR-21 may also have a metabolic function in murine skeletal muscle [46] and in white adipose tissues [47].

Limited information is available for miR-21 in diabetes. Studies in *db/db* mice demonstrated the protective effect of silencing miR-21 on diabetic retinopathy by ameliorating neovascularisation and inflammation [48]. Moreover, in renal mesangial cells exposed to high glucose, miR-21 has been found to decrease PTEN expression [49] and can also influence beta cell apoptosis [50]. Additionally, miR-21 is a critical regulator of hepatic gluconeogenesis and may be a novel therapeutic target for treating insulin resistance and diabetes [51]. Its absence in macrophages increases the expression of the miR-21 target gene MKK3, promoting apoptosis, plaque necrosis, and vascular inflammation during atherogenesis [52].

The effects of ROS on miRNA expression levels and their roles in ROS-mediated gene regulation in vascular cells are the subject of speculation. In our results, oxidative stress, measured by the only technique that provides direct evidence and an absolute quantification of ROS (by EPR), was induced in oscillating and high glucose conditions in HUVECs, and silencing with anti-miR-21 reduced ROS, highlighting the capability of anti-miR-21 to counteract superoxide anion production, as well as to restore the mitochondrial membrane potential.

We demonstrated that the expression of miR-21 is associated with the inhibition of high glucose-mediated KRIT1, ERK and NRF2 signalling, which decreased the expression and activation of SOD2, suppressing antioxidant responses. First, immunostaining assays revealed that KRIT1 induction increased during silencing of miR-21 concomitantly with ERK and NRF2 activation, as shown by their phosphorylated status. KRIT1 is a novel player involved in the maintenance of intracellular vascular ROS homeostasis and in the integrity of endothelial functions and is primarily expressed in endothelial cells. The occurrence of variations in antioxidant defence mechanisms has been demonstrated by KRIT1 genetic knockout technology as well as in various cell types, such as HUVECs [53]. Original findings have indicated that KRIT1 is involved in the maintenance of intracellular ROS homeostasis through the modulation of master regulators of cellular responses to oxidative stress, including FoxO1 and SOD2, which prevent the accumulation of superoxide anions produced at the mitochondrial electron transport chain and consequent mitochondrial and cellular dysfunctions. Our results suggest that the high glucose-responsive miR-21 can regulate the effects of ROS via the KRIT1 pathway. The redox sensitive transcription factor Nuclear Erythroid 2 related factor-2 (NFE2L2 or NRF2) regulates antioxidant response elements (AREs). Decreased NRF2 activity is known to contribute to increasing oxidative stress, mitochondrial dysfunction in vessels, and endothelial dysfunction, as observed in diabetes [54]. NRF2 activation is associated with preventing many types of human diseases, such as diabetes and obesity [55]. Data from Cong et al. demonstrated the endogenous induction of NRF2 by ERK activation [56].

Our analyses identified a miR-21-dependent interfering effect on SOD2 mRNA and protein expression, which affected the scavenging action in OG and HG conditions. Thus, targeting miR-21 could be the mechanism by which KRIT1 regulates ROS homeostasis and improves antioxidant responses by increasing SOD2 expression.

Our studies emphasize the importance of preserving an efficient antioxidant response by inhibiting miR-21, thus avoiding Ox-S and its associated diabetic damage. In addition, the role of a failure in antioxidant enzymes has been highlighted in pancreatic beta cells as well as in tissues such as the liver and kidneys of diabetic mice. The latter findings could be explained by the increased miR-21/ROS axis, which in turn could activate the fibrotic phenomenon and enhance the disease. A low antioxidant defence capacity is considered to be an important aspect of ROS damage leading to beta cell death and endothelial dysfunction. Although stable overexpression of SOD2 in RINm5F and INS-1E cells provided complete protection against cytotoxicity by conferring resistance to oxidative stress damage [57, 58], the use of exogenous antioxidant supplementation in the majority of antioxidant clinical trials has demonstrated limited success and failed to show significant advantages in the prevention among subjects with or without clinical sign of cardiovascular complications [59]. Multiple challenges remain to be identified. Undoubtedly, defective antioxidant systems would be induced among endogenous systems since the use of exogenous antioxidants may be limited by different absorption levels in the intracellular locations of the subjects.

In this work, we showed that the loss of KRIT1, induced by miR-21 in GV and chronic stable high glucose conditions, interferes with SOD2 expression and affects antioxidant response systems, resulting in mitochondrial dysfunction.

## Conclusion

Elevated levels of intracellular miR-21 and ROS generation in HUVECs during oscillating and high glucose exposures suggested a strict relationship between miR-21 and acute hyperglycaemia, as well as a role in the regulation of mitochondrial dysfunction, and it may be considered Ox-S-induced markers of damage.

## Additional file

**Additional file 1: Figure S1.** Transfection efficiencies of the use of anti-miR-21 inhibitor evaluated by (A) viability assay using Trypan blue exclusion dye (dil 1:10), and by (B) q-PCR of miR-21 expression levels.

## Abbreviations

ΔΨm: delta psi mitochondrial
EC: endothelial cells
EPR: electron paramagnetic resonance
ERK 1–2: extracellular signal-regulated kinase 1–2
FOXO1: forkhead box O1
HG: high glucose
KRIT1: krev/rap interaction trapped 1
MMP: mitochondrial membrane potential
NFE2L2: nuclear factor erythroid 2 like 2
NG: normal glucose
OG: oscillating glucose
Ox-S: oxidative stress
ROS: reactive oxygen species
SOD2: superoxide dismutase 2
T2D: type 2 diabetes
