# Supplementary material for: Glucose-sensing microRNA-21 disrupts ROS homeostasis and impairs antioxidant responses in cellular glucose variability
Source: Cardiovasc Diabetol. 2018 Jul 23;17:105. doi: 10.1186/s12933-018-0748-2 (PMC6055345; doi:10.1186/s12933-018-0748-2)
Supplement: Supplementary file 1 — Additional file 1: Figure S1. Transfection efficiencies of the use of anti-miR-21 inhibitor evaluated by (A) viability assay using Trypan blue exclusion dye (dil 1:10), and by (B) q-PCR of miR-21 expression levels. [file 12933_2018_748_MOESM1_ESM.pdf]

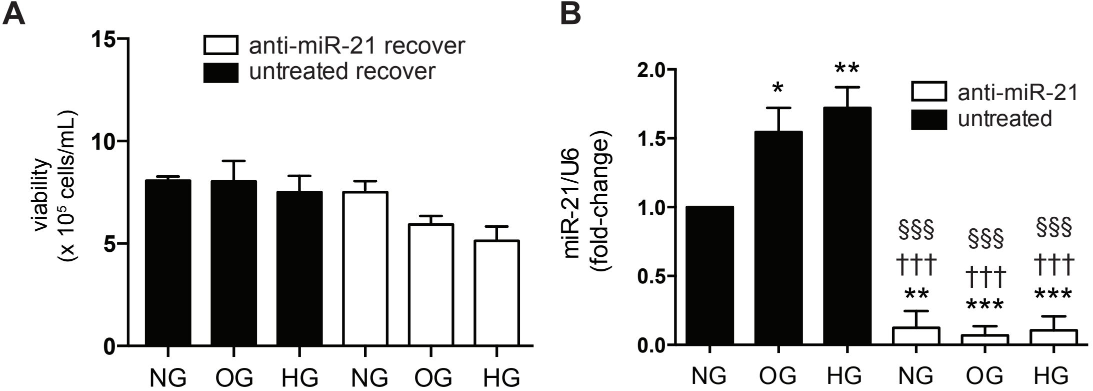

Additional file 1: Figure S1 Transfection efficiencies of the use of anti-miR-21 inhibitor evaluated by (A) viability assay using Trypan blue exclusion dye (dil 1:10), and by (B) q-PCR of miR-21 expression levels.
